# Vasoprotective Effects of Hyperoside against Cerebral Ischemia/Reperfusion Injury in Rats: Activation of Large-Conductance Ca^2+^-Activated K^+^ Channels

**DOI:** 10.1155/2023/5545205

**Published:** 2023-08-14

**Authors:** Wen-Ming Hong, Yue-Wu Xie, Meng-Yu Zhao, Tian-Hang Yu, Li-Na Wang, Wan-Yan Xu, Shen Gao, Hua-Bao Cai, Yan Guo, Fang Zhang

**Affiliations:** ^1^Department of Neurosurgery, First Affiliated Hospital of Anhui Medical University, Hefei 230032, China; ^2^School of Nursing, Anhui Medical University, Hefei 230032, China; ^3^Open Project of Key Laboratory of Dermatology, Ministry of Education, Anhui Medical University, Hefei 230032, China; ^4^School of Pharmacy, Wannan Medical College, Wuhu 241002, China; ^5^Department of Pharmacology, Anhui Medical University, Hefei 230032, China

## Abstract

Hyperoside (Hyp), a kind of Chinese herbal medicine, exerts multiple therapeutic effects on many diseases. However, the role and mechanisms of Hyp in vascular pathophysiology in ischemic stroke need to be further established. The study aimed to investigate the role of (large-conductance Ca^2+^-activated K^+^) BK channels on the vasoprotection of Hyp against cerebral ischemia and reperfusion (I/R) injury in rats. The concentration gradient of Hyp was pretreated in both the middle cerebral artery occlusion and reperfusion model and oxygen-glucose deprivation/reoxygenation (OGD/R) model of primary vascular smooth muscle cells (VSMCs) in rats. A series of indicators were detected, including neurological deficit score, infarct volume, malondialdehyde (MDA), superoxide dismutase (SOD), cerebral blood flow (CBF), cell viability, membrane potential, and BK channels *α*- and *β*1-subunits expression. The results showed that Hyp significantly reduced infarct volume and ameliorated neurological dysfunction in I/R-injured rats. Besides, the effects of I/R-induced reduction of BK channels *α*- and *β*1-subunits expression were significantly reversed by Hyp in endothelial-denudated cerebral basilar arteries. Furthermore, the protective effect against I/R-induced increases of MDA and reduction of SOD as well as CBF induced by Hyp was significantly reversed by iberiotoxin (IbTX). In OGD/R-injured VSMCs, downregulated cellular viability and BK channels *β*1-subunits expression were remarkably reversed by Hyp. However, neither OGD/R nor Hyp affected BK channels *α*-subunits expression, and Hyp failed to induced hyperpolarization of VSMCs. Moreover, the protective effect against OGD/R-induced reduction of cell viability and SOD level and increases of MDA production induced by Hyp was significantly reversed by IbTX in VSMCs. The study indicates that Hyp has the therapeutic potential to improve vascular outcomes, and the mechanism is associated with suppressing oxidative stress and improving CBF through upregulating BK channels.

## 1. Introduction

An ischemic stroke is a complicated and devastating event initiated by cerebrovascular disorder, followed by a series of nerve degenerative changes [1]. Up until now, the most effective treatment is rapid recanalization of the occluded vessel by recombinant tissue plasminogen activator [2]. However, reperfusion after a long period of ischemia may bring about secondary damage, which is termed cerebral ischemia and reperfusion (I/R) injury [3]. As a cerebrovascular disease, dysfunction of the vascular smooth muscle (VSM) in the macro- and micro-vascular is regarded as one of the fundamental causes leading to impaired perfusion and subsequently to impairment of tissues [4]. Pharmacological interventions for ischemic stroke are needed; however, very few drugs have triumphantly made it through clinical trials [5]. Besides, the entirety interpretation of the potential mechanism is still lacking, especially the vascular pathophysiology.

Hyperoside (Hyp), also known as quercetin-3-O-*β*-D-galactoside pyranose, is a bioactive flavonol glycoside compound primarily isolated from *Hypericum* and *Crataegus* species. Accumulated studies have validated its therapeutic effects on myocardial ischemia [6], tumor progression [7], hepatitis, and pain in various cellar and animal models [8]. Involved mechanisms include the suppression of inflammatory response [9], the downregulation of intracellular calcium level [10], the enhancement of an antioxidative defense system [11], as well as inhibition of proapoptosis [12]. Our previous report demonstrated that Hyp could dominantly relax isolated abdominal aortic rings in an endothelium-dependent manner, and similar relaxation was found in brain middle cerebral artery (MCA) and basal arteries (BA) rings in rats [13]. However, the involved mechanism, which is responsible for its vascular active properties, remains vague.

It is well established that ion channels play a significant role in modulating vascular reactivity, and healthy vasculature is significantly related to benign brain function [14, 15]. The Ca^2+^-activated K^+^ channel family consists of small-, intermediate-, and large-conductance Ca^2+^-activated K^+^ channels (SK, IK, and BK, respectively) [16]. Larger conductance Ca^2+^-activated K^+^ channels (BK, BKca, and MaxiK), which is the major mediator of vascular tension in vascular smooth muscle cells (VSMCs), acts as a final target of the vasodilators [17]. Structurally, the basic functional subunits of BK channels are the pore-forming *α*-subunit encoded by a single gene (Slo, KCNMA1) and four assistant *β*-subunits (*β*1-4) contributed to the channel's molecular diversity [18]. Among the assistant *β*-subunits, *β*1-subunit is highly expressed and is predominant in smooth muscle, while the expression levels of *β*2–4 are negligible [19]. Alterations of expression and/or activity of BK channels are linked to a series of diseases resulted from vascular dysfunction, such as hypertension [20], diabetes [21], and metabolic syndrome [20]. It has been reported that novel and selective BK channels opener NS11021 could inhibit the contractility of urinary bladder smooth muscle in pigs [22]. As a flavonoid compound, baicalin can induce mesenteric arteries relaxation through activating BK channels in rats [23]. We previously found that Hyp induced hyperpolarization of VSMCs in BA, thereby resulting in vascular relaxation, and this effect was significantly suppressed by tetraethylammonium (a nonselective blocker of Ca^2+^-activated K^+^ channels) [13]. However, we did not clarify which channel subtype was responsible for this effect.

The following study aimed to explore the vasoprotective effect of Hyp in experimental stroke models *in vivo* and *in vitro*. The models included middle cerebral artery occlusion (MCAO) and reperfusion, and oxygen-glucose deprivation/reoxygenation (OGD/R) in primary culture VSMCs in rats. Meanwhile, we examined whether BK channels were involved in the vasoprotective effect induced by Hyp pretreatment.

## 2. Materials and Methods

### 2.1. Animals

Male Sprague–Dawley rats, 6–8 weeks of age and ranging in weight from 250 to 280 g, purchased from the Experimental Animal Center of Anhui Medical University and maintained in standard conditions: 22 ± 3°C; 50% humidity; a 12 hr light/dark cycle with free access to chow and water. The following investigation was carried out according to regulations of the Animal Care Committee of Anhui Medical University (Hefei, China; Approval number: LLSC, 20201009) and was consistent with the National Institutes of Health Guide (Publication No. 85-23, revised 2011) for the Care and Use of Laboratory Animals. All efforts were made to minimize the pain of the animal, while animal ethics were strictly followed.

### 2.2. Materials

Hyp (purity > 99%) was obtained from Chengdu Must Biotechnology Co. Ltd. (Chengdu, China; Lot# MUST-18100910). As a BK channels-specific blocker, iberiotoxin (IbTX) was purchased from TOCRIs Bioscience (Bristol, UK). Malondialdehyde (MDA) and superoxide dismutase (SOD) assay kits were obtained from Jiancheng Bioengineering Institute (Nanjing, China). Radioimmunoprecipitation (RIPA) lysis buffer and BCA Protein Quantitation Kit were acquired from the Beyotime Institute of Biotechnology (Shanghai, China). The monovalent anionic voltage-sensitive dye named bis-1,3-dibutylbarbituric acid-trimethine oxonol (DiBaC4(3)) was obtained from Abcam Trading Co., Ltd. (Shanghai, China). Primary antibodies against BK *α*-subunit (catalog no. ab192759) and BK *β*1-subunit (catalog no. ab3587) were obtained from Abcam (Cambridge, UK). Antibodies against *α*-smooth muscle actin (*α*-SMA) (catalog no. 67735-1-Ig) and *β*-actin (catalog no. 66009-1-Ig) were purchased from Proteintech Group (Wuhan, China). Relative secondary antibodies were purchased from Zhongshan Golden Bridge Biotechnology (Beijing, China). All the other chemical reagents, except special instructions, were from Sigma–Aldrich (St. Louis, MO, USA).

### 2.3. MCAO Surgery and Drug Administration

The MCAO experiments were performed as previous descriptions with a few modifications [24]. All rats were anesthetized with 5% isoflurane, and anesthesia was maintained with 3% isoflurane on a ventilator. The rectal temperature was maintained at 37.0 ± 0.5°C with a heating pad throughout the I/R procedure. Basically, the MCAO model was established by inserting a single nylon structure (Cinontech, Beijing, China) with a blunt end at the tip through the right common carotid artery into the internal carotid artery and continued for another 15 mm until the origin of MCA was blocked. In order to induce I/R injury, the filament was left in place for 2 hr of ischemia, followed by removing out for subsequent 22 hr of reperfusion. Rats in the sham group experienced the same operation procedure except the insertion of nylon line. Postsurgery intramuscular penicillin G (25,000 U/kg) was administered to prevent infection. The health of rats was verified by observing the skin, activity, food intake, excretion, abdominal respiration, external genitalia, and eyes following surgery. Afterward, the rats were sacrificed under anesthesia once most of the parameters were abnormal. The rats were considered to be dead when we were unable to detect their breath and heartbeat for more than 3 min.

**Figure 1:**
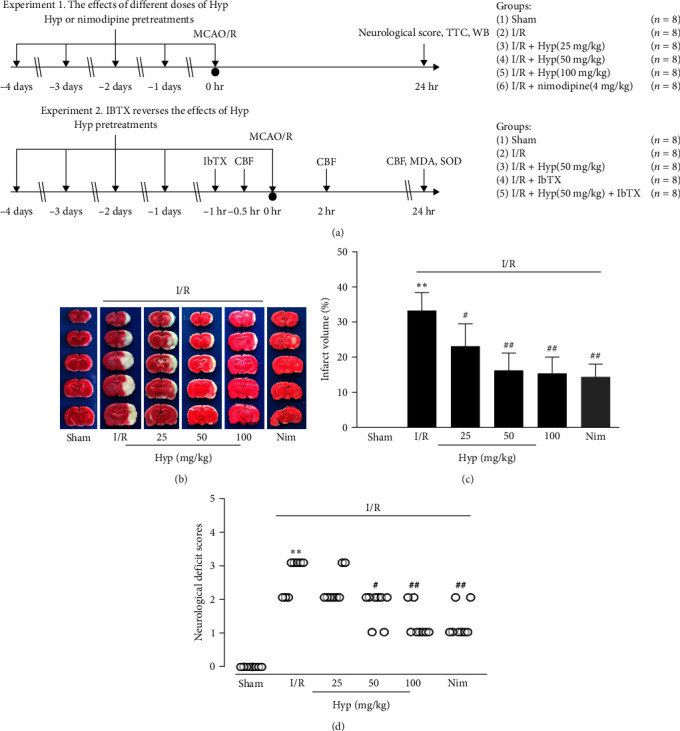
Effect of Hyp on neurological deficit and cerebral infarct volume of I/R-injured rats. Rats were subjected to 2 hr of middle cerebral artery occlusion, followed by 22 hr of reperfusion. (a) Experimental design and animal grouping. (b) Representative images of TTC staining photographs were shown. (c) Quantitative analysis of cerebral infarct volumes was shown. (d) Neurological deficit scores were evaluated according to the Longa EZ graded scoring system. Data are mean ± SD (*n* = 6).  ^*∗∗*^*P* < 0.01 versus sham group; ^#^*P* < 0.05, ^##^*P* < 0.01 versus I/R group.

The *in vivo* study consists of two sets of different experiments (Figure 1(a)). One was the dose-dependent experiment; rats were randomly divided into the following six groups (*n* = 8): sham group, I/R group, 25, 50, and 100 mg/kg Hyp-pretreatment groups, and 4 mg/kg nimodipine group. In order to detect a possible role of the BK channels in the vasoprotective effect of Hyp, rats were randomly assigned to five groups (*n* = 8): sham group, I/R group, 50 mg/kg Hyp group, IbTX group, 50 mg/kg Hyp + IbTX group. Indicated concentrations of drug were administrated intraperitoneally once a day for 5 days. In the present study, the rationale behind the selection of Hyp dose as well as the administration routes was based on previous studies [25–27]. Hyp was dissolved in dimethyl sulfoxide (DMSO) solution and then diluted with 0.9% saline just before injection. The final concentration of DMSO used in rats was <1%. Moreover, the same volume of 0.9% saline was injected intraperitoneally in the sham group at the corresponding time point. Nimodipine was used as a comparative positive control [28]. To explore the role of BK channels in the protective effects of Hyp against I/R injury in rats, IbTX (250 nmol/kg) was used to block BK channels at 1 hr before MCAO based on our previous study with a few modifications [29]. Nimodipine and IbTX were dissolved in 0.9% saline just before injection.

### 2.4. Neurological Score and Measurement of Cerebral Infarct Volume

A neurological deficit score was performed immediately after cerebral I/R injury according to a modified Longa EZ graded scoring system [30]: 0, no deficit; (1) endoduction and incomplete extension in the left forelimb; (2) circling to the paralytic side; (3) falling to the contralateral side of the affected brain; (4) did not walk on its own accompanied by disorders of consciousness. To determine the infarct volume, the whole brain was quickly removed out and placed in the freezer for 20 min. Then the brain samples were sectioned into 2 mm-thick coronal slices and stained with the prepared 2% 2,3,5-triphenyltetrazolium chloride (TTC) for 20 min at 37°C in dark. After being fixed overnight in 4% paraformaldehyde (pH = 7.4) at 4°C, the brain sections were photographed and qualified by Image J software. The infarct volume was calculated using a percentage of the whole brain volume according to a previous study [31].

### 2.5. Laser Speckle Contrast Imaging (LSCI)

LSCI of regional cerebral blood flow (CBF) in the cortex was performed during the surgery by a laser speckle contrast analysis imager (Perimed, Järfälla, Sweden). After anesthetized, the skull of each rat was exposed and thinned after a midline scalp cut, and CBF was monitored by a charge-coupled device camera adjusted 10 cm above the skull through its assistant arm. All CBF measurements were obtained through two identical regions of interest (ROI) over the right and left MCA. Raw laser speckle images (16 pictures per second) and relative data were collected. In order to obtain mean CBF flux, CBF flux in each rat was calculated of 3 min of baseline period before I/R injury, 3 min of intraischemic period before moving out of the filament, and 3 min of postreperfusion period before the end of I/R injury. The relative change of CBF flux in the ROI was normalized to a percentage of preischamic baseline values.

### 2.6. Cell Culture

Primary rat cerebral basilar artery VSMCs were acquired by means of the wall-adherence method. Briefly, the whole brain tissues harvested by decapitation were immersed in a dish filled with cold physiological salt solution (PSS) under sterile conditions. PSS contained (in mM) 137 NaCl, 5.6 KCl, 1 MgCl_2_, 0.42 Na_2_HPO_4_, 0.44 NaH_2_PO_4_, 4.2 NaHCO_3_, and 10 HEPES (pH 7.4). The BA were gently peeled off and freed of connective tissues, and endothelium was rubbed off with a small stainless-steel wire under a stereoscope (Olympus, Tokyo, Japan). Then minced tissues were cultured at 37°C and 5% CO_2_ after supplementing with 4–6 mL 20% fetal bovine serum-containing Dulbecco's Modified Eagle's Medium (DMEM) (Hyclone, South America). Media was renewed every 3 days, and passages of 3–6 were used after overnight serum starvation in the subsequent experiments.

### 2.7. Immunofluorescent Staining

In order to identify primary VSMCs, immunostaining was performed as previously described [32]. After fixation, permeabilization, and blocking, VSMCs were then incubated with an anti-*α*-SMA antibody for 24 hr at 4°C followed by immersing in Alexa Fluor 488-conjugated secondary antibody (1 : 1,000) for 1 hr at room temperature. DAPI was used to stain the cell nucleus for 5 min. Then stained slides were examined under an inverted microscope (Zeiss, Germany).

### 2.8. OGD/R and Experimental Protocol

To simulate I/R injury, an *in vitro* OGD/R model was established. Cell culture medium of treatment groups was changed into Krebs-Ringer-HEPES buffer containing (in mM) 115 NaCl, 1 CaCl_2_, 5 KCl, 1 KH_2_PO_4_, 1.2 MgSO_4_·7H_2_O and 25 HEPES (pH 6.7), then subjected to an anaerobic environment formed by saturating with 94% N_2_-5% CO_2_ in a special three-gas incubator (Heraeus, Germany). After 4 hr, plates with renewed medium of DMEM were transferred into the normal cell incubator for another 20 hr. In the meantime, indicated concentrations of Hyp and nimodipine were added into the culture medium for pretreatment. The control group did not experience any operations except renewing the medium with DMEM synchronously. The concentrations of Hyp were 10, 33, and 100 *μ*M, and nimodipine was 10 *μ*M. To probe the role of the BK channels in the protective effect of Hyp *in vitro*, VSMCs were randomly assigned to five groups: control group, OGD/R group, 33 *μ*M Hyp group, IbTX group, 33 *μ*M Hyp + IbTX group. IbTX (100 nM) was used during OGD/R based on our previous research [29]. As a vehicle, the volume of DMSO was under 1‰.

### 2.9. MTT Assay

Cell viability was analyzed with an MTT assay. About 6 × 10^3^ cells per well were seeded in 96-well flat-bottomed plates and then incubated in a humidified 5% CO_2_ equilibrated incubator at 37°C for 24 hr. After the molding and dosing process, 10 *μ*L MTT solution (5 mg/mL in PBS) was added into all working wells. The formazan crystals were dissolved with 150 *μ*L DMSO after 4 hr cultivation. Absorbance was read at 490 nm by a Microplate Reader.

### 2.10. Membrane Potential Measurement

VSMCs were seeded evenly on glass coverslips and allowed to reach 80% confluence. To monitor the effect of Hyp on VSMCs membrane potential changes, 2 *μ*M DiBaC4(3) fluorescence dye was added into the medium and incubated at 37°C for 10 min in the dark. Following labeling, slides were washed and fixed in the chamber filled with 1 mL normal PSS (N-PSS), which contained (in mM) NaCl 140, KCl 5, MgCl_2_ 1, CaCl_2_ 2, D-Glucose 10, HEPES 10. Membrane potential variation was probed by using a fluorescence microscope equipped with Metafluor software (Eclipse Ti, Nikon, Tokyo, Japan). Individual cells were selected and imaged with a stimulated wavelength of 488 nm and an emission wavelength of 530 nm. The stable fluorescence was acquired after 5–10 min' equilibration. The intensity of changes in green fluorescence represented the variation of membrane potential. The results were presented as a percentage of the ratio of instantaneous fluorescence intensity value to the baseline after subtraction of the background.

### 2.11. MDA and SOD Assay

To determine the MDA and SOD level, the homogenate of endothelial-denudated BA or cell supernatant were subjected to various treatments in strict accordance with the protocols of the MDA and SOD assay kits. Changes in absorbance were read at their respective wavelength. Experiments were performed in triplicate.

### 2.12. Western Blots

Western blotting experiments were performed according to a previously standardized protocol [33]. The endothelial-denudated BA was cut into small pieces and homogenized on ice. Briefly, vascular and cellular lysates were obtained using the ice-cold RIPA buffer with 1% phenyl-methanesulfonyl fluoride and 1% protease inhibitor cocktail. Equivalent protein samples were subjected to 10% Sodium dodecyl-sulfate polyacrylamide gel electrophoresis and transferred onto a polyvinylidene fluoride membrane (Millipore, Bedford, MA, USA). After blocking, these membranes were incubated with primary antibodies and respective secondary antibodies. Immunoreactive proteins were detected using the enhanced chemiluminescence method. Lanes of interest were normalized to the *β*-actin internal control.

### 2.13. Statistical Analysis

Data were expressed as mean ± standard deviation (SD). Statistical analysis was performed using one-way analyses of variance, followed by the Student–Newman–Keuls test. The neurological deficit scores were analyzed by the Kruskal–Wallis test followed by Mann–Whitney *U*-test with the Bonferroni correction. *P* < 0.05 was considered to be statistically significant.

## 3. Results

### 3.1. Hyp Attenuates Neurological Deficit and Infarct Volume

In *in vivo* experiments, a total of 101 rats fulfilled the model establishment (101 were used; 88 survived). Among the dead rats, one died due to the sham-operation, and 12 deaths occurred in the I/R group, with a mortality rate of 14.3% (12/84 rats). The success of the MCAO model was evident, as the I/R-injured rats exhibited an obvious increase in neurological deficit scores. Intraperitoneal injection of the positive control drug nimodipine or different doses of Hyp for 5 days in advance significantly decreased the neurological deficits compared to the I/R group (Figure 1(d)). Representative TTC-stained cortex brain sections are shown in Figure 1(b) MCAO injury caused a significant increase in infarct volume in the I/R group, while the sham group showed no infarct area. Compared to the I/R group, Hyp 25, 50, and 100 mg/kg groups significantly reduced infarct volume to 23.4% ± 6.5%, 16.4% ± 5.0%, and 15.6% ± 4.7%, respectively. Besides, brain infarction induced by I/R was also significantly prevented by nimodipine (Figure 1(c)). These data suggested that Hyp has significant neuroprotective effects against I/R injury.

### 3.2. Hyp Significantly Upregulates BK *α*- and *β*1-Subunits Expression in I/R-Injured Cerebral BA

**Figure 2:**
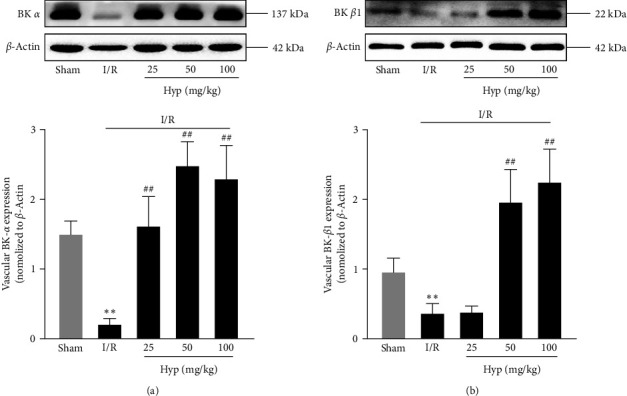
Effect of Hyp on BK *α*- and *β*1-subunits expression in I/R-injured cerebral basilar arteries in rats. The expression and analysis of BK *α*-subunits (a) and BK *β*1-subunits (b) in I/R-injured cerebral basilar arteries. Data are mean ± SD (*n* = 5).  ^*∗∗*^*P* < 0.01 versus sham group; ^##^*P* < 0.01 versus I/R group.

Western blotting was used to elucidate the underlying mechanism involved in the effects of Hyp on I/R-injured cerebral vessels. And the results showed that both BK *α*- and *β*1-subunits expression was significantly decreased in I/R-injured cerebral BA, while they were both notably reversed by Hyp pretreatment (Figures 2(a) and 2(b)).

### 3.3. IbTX Reversed the Protective Effects of Hyp against I/R-Induced Oxidative Stress in Cerebral BA

**Figure 3:**
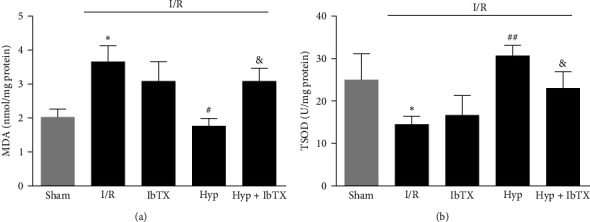
Effects of iberiotoxin on the protective effects of Hyp against I/R-induced oxidative stress in cerebral basilar arteries. (a) MDA levels; (b) SOD activities. Data are mean ± SD (*n* = 5).  ^*∗*^*P* < 0.05 versus sham group; ^#^*P* < 0.05, ^##^*P* < 0.01 versus I/R group; ^&^*P* < 0.05 versus I/R + Hyp group.

The level of MDA was dramatically elevated, while the SOD level was obviously decreased in I/R-injured cerebral BA compared with those in sham groups. Pretreatment with Hyp (50 mg/kg) significantly decreased the MDA level and promoted the SOD activities, while these effects were reversed by IbTX (Figures 3(a) and 3(b)). The results suggest that Hyp could suppress oxidative stress and improve the antioxidant capacity of cerebral blood vessels through upregulating the BK channels.

### 3.4. IbTX Reversed the Hyp-Induced Protective Effects of CBF during I/R in Rats

**Figure 4:**
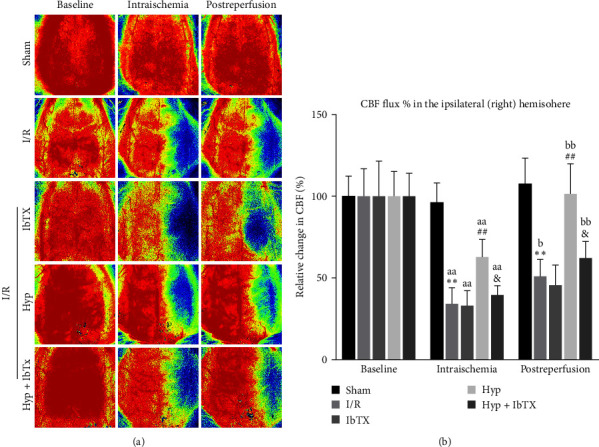
Effects of iberiotoxin on Hyp-induced protective effects of CBF during I/R in rats. (a) Representative pictures of laser speckle contrast imaging of CBF in the cortex in rats submitted to I/R injury. The baseline CBF was measured just before I/R injury. (b) The regional CBF in the ipsilateral (right) hemisphere in I/R-injured rats. Data were expressed as a percent of baseline values. Mean ± SD, *n* = 5/group.  ^*∗∗*^*P* < 0.01 versus sham group; ^##^*P* < 0.01 versus I/R group; ^&^*P* < 0.05 versus I/R + Hyp group. ^aa^*P* < 0.01 versus baseline group; ^b^*P* < 0.05, ^bb^*P* < 0.01 versus intraischemia group.

As shown in Figure 4(a), in these laser speckle contrast images with pseudo color, the right cortex rectangular ROI of MCAO model, which reflected as blue, represented a low level of blood flow. We reported that regional CBF for each rat was normalized to its respective baseline (percentage baseline of CBF). The CBF flux in the sham group showed no significant changes during I/R periods. Intraischemically, the CBF flux in the ipsilateral MCA territory was declined to 34.3% ± 9.8% from the baseline and further gradually recovered to 51.0% ± 10.3% after 22 hr of reperfusion. IbTX had no significant effect on the CBF flux during I/R injury. Pretreatment of Hyp at a dose of 50 mg/kg significantly improved the CBF flux at both I/R time points in the ipsilateral hemisphere. However, these protective effects were significantly reversed by IbTX pretreatments (Figure 4(b)).

### 3.5. Hyp Alleviated OGD/R-Induced Injury in Primary VSMCs

**Figure 5:**
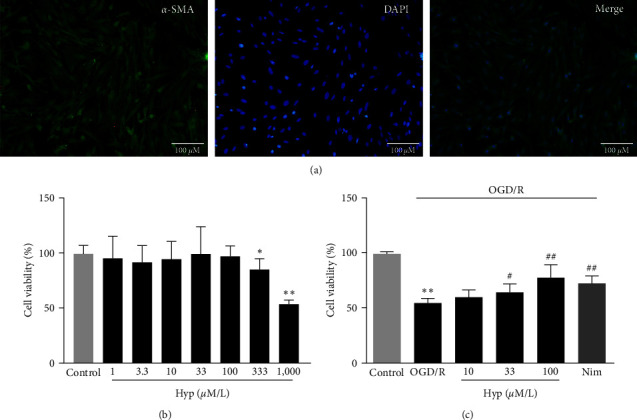
Effects of Hyp on OGD/R-induced injury in primary VSMCs. (a) Primary VSMCs were identified by immunofluorescence and morphology. Scale bars = 100 *µ*m. (b) VSMCs were coincubated with various concentrations of Hyp (1–1,000 *μ*M) for 24 hr, and cytotoxicity was detected by an MTT assay. (c) VSMCs were pretreated with Hyp (10, 33, and 100 *μ*M) or nimodipine (10 *μ*M) during OGD/R, and cell viability was calculated by an MTT method. Data are mean ± SD (*n* = 5).  ^*∗*^*P* < 0.05,  ^*∗∗*^*P* < 0.01 versus control group; ^#^*P* < 0.05, ^##^*P* < 0.01 versus OGD/R group.

Morphological analysis revealed that primary VSMCs exhibited a long spindle shape with a typical “hill-and-valley” pattern. Cells of the second passage were stained with anti-*α*-SMA FITC (green) and DAPI (blue), the cytoplasm and nucleus were then presented in green fluorescence and blue fluorescence, respectively. The results demonstrated that the cells were indeed VSMCs, and the purity was nearly 100% (Figure 5(a)). To evaluate the cytotoxicity of the compound, different concentrations of Hyp (from 1 to 1,000 *μ*M) were co-incubated with VSMCs for 24 hr. As shown in Figure 5(b), the concentrations of Hyp under 100 *μ*M had no cytotoxicity, while a significant inhibition of cell activity was found when the concentrations exceeded 333 *μ*M. Hence, the concentrations (10, 33, and 100 *μ*M) were selected for subsequent experiments. As shown in Figure 5(c), cell viability decreased to 55.0% ± 4.2% of the control after exposing to OGD/R; however, it was restored by nimodipine and Hyp pretreatment dose-dependently. Therefore, these results suggested that Hyp pretreatment could increase viability in VSMCs.

### 3.6. Hyp Selectively Upregulates BK *β*1-Subunit but Not *α*-Subunit Expression in OGD/R-Injured VSMCs

**Figure 6:**
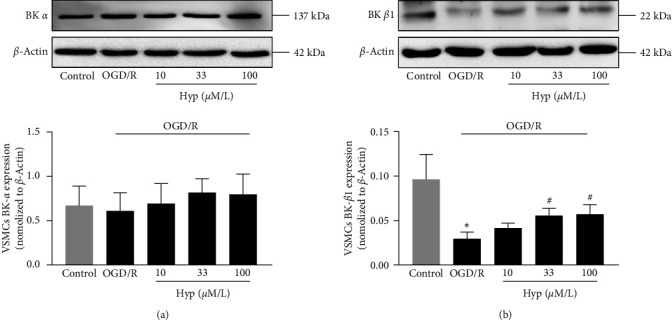
Effects of Hyp on BK *α*- and *β*1-subunits expression in OGD/R-injured VSMCs. The expression and analysis of BK *α*-subunit (a) and BK *β*1-subunit (b) in OGD/R-injured VSMCs. Data are mean ± SD (*n* = 5).  ^*∗*^*P* < 0.05 versus control group; ^#^*P* < 0.05 versus OGD/R group.

BK channel is the predominant type of ion channels in VSMCs [19]. Next, we investigated whether BK channels were involved in Hyp-induced protection against OGD/R in VSMCs. As shown in Figure 6(a), there was no significant difference in the BK *α*-subunit expression in each group. However, the BK *β*1-subunit expression was significantly decreased after OGD/R injury, and pretreatment with Hyp markedly reversed this effect (Figure 6(b)). The results showed that Hyp could selectively upregulate BK channel *β*1-subunit, not *α*-subunit in VSMCs subjected to OGD/R injury.

### 3.7. Hyp Fails to Induce Membrane Potential Hyperpolarization of VSMCs

**Figure 7:**
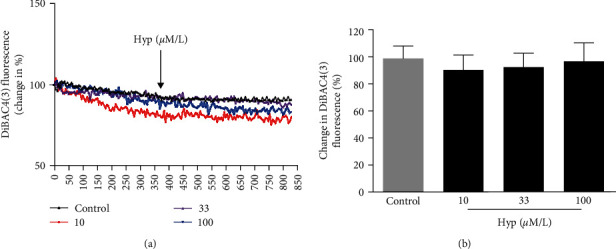
Effects of Hyp on the membrane potential of VSMCs. VSMCs were treated with Hyp after loading with DIBAC_4_ (3), and changes in membrane potential were detected. (a) Representative traces for changes in membrane potential in response to Hyp were shown. (b) Quantitative analysis of membrane potential in response to Hyp was shown. Data are mean ± SD (*n* = 5).

The VSMCs membrane potential acts as a crucial role in maintaining vascular tone [34]. Accumulated evidence suggests that membrane potential plays an important role in regulating the contraction of VSMCs. Activation of BK channels would induce hyperpolarization in VSMCs, deactivate voltage-gated Ca^2+^ channels, and limit their excessive contraction [35]. To further explore the role of Hyp in modulating BK channels, changes in membrane potential in VSMCs were assessed using the fluorescence probe DiBaC4(3). As shown in Figure 7, the fluorescence intensity ratio for Hyp at the dose of 10, 33, and 100 *μ*M were 91. 2% ± 11.3% (*P* > 0.05 compared to the control), 93.4% ± 10.5% (*P* > 0.05 compared to the control) and 97.8% ± 13.8% (*P* > 0.05 compared to the control), respectively. The results indicate that Hyp could not activate BK channels in VSMCs.

### 3.8. IbTX Reversed the Protective Effect of Hyp against OGD/R-Induced Oxidative Stress in VSMCs

**Figure 8:**
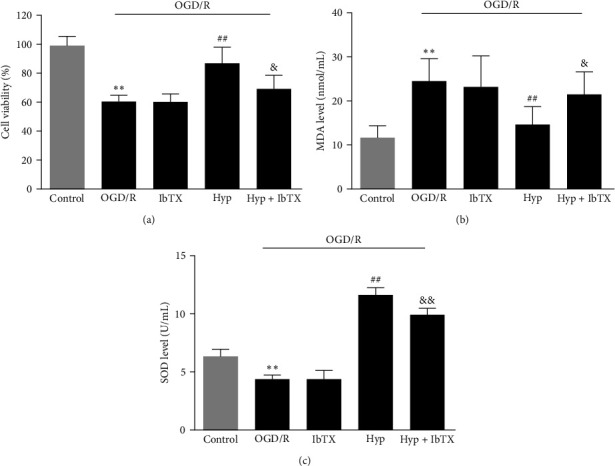
Effects of iberiotoxin on the protective effect of Hyp against OGD/R-induced oxidative stress in VSMCs. (a) Cell viability by an MTT method; (b) MDA contents; (c) SOD activities. Data are mean ± SD (*n* = 5).  ^*∗∗*^*P* < 0.01 versus control group; ^##^*P* < 0.01 versus OGD/R group; ^&^*P* < 0.05, ^&&^*P* < 0.01 versus OGD/R + Hyp group.

As shown in Figure 8(a), cell viability was significantly decreased after OGD/R, whereas Hyp (33 *μ*M) significantly enhanced the cell activity. Besides, the MDA level was significantly increased after OGD/R, whereas Hyp remarkedly inhibited the production of MDA. Moreover, pretreatment with Hyp notably reversed the reduced SOD levels in OGD/R-injured VSMCs (Figures 8(b) and 8(c)). Pretreatment of IbTX had no significant effects on OGD/R-injured VSMCs; however, it could significantly reverse the protective effects of Hyp against OGD/R-induced injury. Therefore, these results demonstrated that Hyp suppressed oxidative stress in OGD/R-injured VSMCs through activating BK channels.

## 4. Discussion

The present study was undertaken to determine whether pretreatment with Hyp had vasoactive effects in rats undergoing cerebral I/R injury. And we demonstrated for the first time that Hyp exerted cerebrovascular protection against ischemic stroke by upregulation of vascular BK channels.

In clinics, cerebral ischemia injury has become an important cause of morbidity and mortality, and disability worldwide [36]. Under the terrible pathological condition, few neuroprotective treatments could work very well due to the injured cerebral vasculature. Hence, any therapeutic approaches that aim to improve the poor cerebral vasculature and explore the potential mechanism would be desperately needed. A growing body of studies has been performed to investigate the therapeutic potential of the traditional herb [37]. Previous research has reported that a diet rich in flavonoids has protection effects against cardiovascular I/R injury through various pathways [38]. As a flavonoid widely present in Chinese herbs, it has been reported that pretreatment of Hyp by intragastrical administration for 5 days could obviously improve cerebral infarction and neurological deficits in rats subjected to focal cerebral I/R injury [39]. It has been found that when the dose of Hyp was higher than 1,000 mg/kg would have certain toxicity during embryonic/fetal development in rats [40]. Therefore, our dosing level was safe. Consistently, pretreatment of Hyp reduced infarct volume and ameliorated neurological dysfunction in I/R-injured rats.

In our study, we focused on the vasoprotective effect of Hyp against ischemic brain injury. As a therapeutic target, BK channels are ubiquitously expressed in VSMCs and play an important role in regulating vascular function, such as vasoconstriction, vascular tone, and membrane potential [22]. Deletion of BK channels *α*-subunit is correlated with a complicated phenotype, such as hyperaldosteronism, high systemic blood pressure, and low serum K^+^ levels [41]. It is widely accepted that *β*1-subunit is primarily expressed in VSM tissues and enables BK channels to respond to Ca^2+^ sparks effectively; therefore, it is important for the vasomotor administration. The reduced sensitivity to calcium signals, abnormal Ca^2+^ Spark/STOC coupling, and increased arterial tone was observed in *β*1 gene knockout or disrupted mice [42]. In pregnant sheep exposed to prolonged hypoxia, downregulation of BK channel *β*1-subunit expression and the BK channel activity was observed in uterine arteries [43]. In one study of patients with partial bladder outlet obstruction and associated detrusor overactivity, both BK *α*- and *β*-subunits were significantly reduced in detrusor smooth muscle [44]. Accumulated evidence suggests that ion channel activity could be modulated by flavonoids, resulting in changes in vascular tone [45]. A recent study by Ye et al. [35] has demonstrated that isoliquiritigenin could induce vasodilation of mouse mesenteric arteries through activating BK channels. In another study, BK channels and Ca-L channels were proved to play an important role in the vasodilatory effect of hydroxy-safflor yellow A [46]. Here, our results revealed that Hyp pretreatment significantly reversed the downregulated expression of BK *α*- and *β*1-subunits induced by experimental stroke in cerebral BA. This study is an extension of our previous findings that Hyp could induce hyperpolarization of VSMCs in BA, identifying Hyp as the activator of BK channels in cerebral BA.

Oxidative stress has been confirmed to be one of the major risk factors of vascular dysfunction and neurodegenerative diseases [47]. Investigators have demonstrated that BK channels are sensitive to the redox environment in many types of tissues and cells. Furthermore, oxidative stress could impair K^+^ channel function in large cerebral and coronary arteries [48]. As a BK channel opener, NS11021 protects the astrocytes from oxidative stress induced by H_2_O_2_ [49]. It has been largely reported that one of the multiple biological activities of flavonoids is that they could maintain vascular health by their opposite interaction with oxidant stress [50]. Our previous research suggested that total flavones of *Rhododendron simsii* planch flower protected hippocampal neurons against hypoxia/reoxygenation injury through activation of BK channels [51]. A *in vivo* study revealed that Hyp could protect cerebral I/R injury by inhibiting oxidative stress, inflammation, and apoptosis in rats, and the potential mechanism is related to the activation of the PI3K/AKT pathway [52]. IbTX, a scorpion venom peptide, has been used to inhibit BK channel with affinity and specificity in mammalian systems [53]. Consistent with their results, we demonstrated that pretreatment of Hyp diminished MDA production and improved SOD activity in cerebral BA, indicating that Hyp has considerable scavenging ability toward vascular oxidative stress. While this protective effect could be remarkably restrained by IbTX, indicating that Hyp protected against vascular oxidant stress through activating BK channels.

The fate of the mammalian brain is critically dependent on an appropriate CBF, which is regulated by a series of coordinated reactions of intricate cerebral blood vessels [54]. It is necessary for effective administration of CBF by functional brain vessels, for sustained and substantial reduction of CBF would lead to a cascade of life-threatening complications resulted from disrupted supply of oxygen and nutrients. Several lines of evidence suggest that activation of BK channels acted as an important role in flavonoids-induced vasorelaxation [46, 55]. Consistently, the present results showed that pretreatment of Hyp significantly improved the CBF flux during I/R in the ipsilateral hemisphere, suggesting the vasoprotective role of Hyp in cerebral blood vessels. Its vasoprotective effect could be significantly attenuated by IbTX, indicating that stimulation of BK channels in cerebral arteries partly contributes to the Hyp-induced increases of CBF.

As a predominant component of the wall vessels, VSMCs play an important role in regulating vascular tone by modulating their diameter, especially in medium and larger arteries [56]. Hence, we set out to investigate the role of Hyp in primary VSMCs subjected to OGD/R injury. We demonstrated that Hyp pretreatment significantly improved the VSMCs viability. It has been suggested that BK channels *β*1-subunit could facilitate unique and effective modulation of channel function against oxidative stress [21]. In type 2 diabetes, BK channels *β*1-subunit were down-regulated and acted as a downstream target of Nrf2, which is the master regulator of the antioxidant response [57]. One study demonstrated that hypoxia suppressed the BK *β*1-subunits expression in human arterial smooth muscle cells, resulting in a decrease of vasodilation [58]. Consistent with the *in vivo* results, we showed that OGD/R suppressed the expression of the BK *β*1-subunits in primary VSMCs, while this effect was significantly reversed by Hyp pretreatment. Moreover, we proved that Hyp suppressed oxidative stress in OGD/R-injured VSMCs, and these effects were markedly restrained by IbTX. These results suggested that Hyp protected VSMCs against OGD/R-induced oxidative stress through up-regulation of the BK *β*1-subunits expression.

In the present study, we found that neither OGD/R nor Hyp could affect the expression of BK channels *α*-subunits in VSMCs. It is suggested that activation of BK channels enables K^+^ efflux and membrane hyperpolarization in VSMCs, resulting in vasodilation. In order to clarify whether Hyp activates BK channels in VSMCs, we performed membrane potential measurements. However, we found Hyp failed to induced hyperpolarization of primary VSMCs, indicating that Hyp could not activate BK channels in VSMCs. These data were inconsistent with our former study that Hyp could induce hyperpolarization of VSMCs in isolated BA in rats [13]. The discrepancy might be due to the different cellular environments in which VSMCs exist. There are close interactions between endothelial cells (ECs) and VSMCs, and their crosstalk plays an essential role in vascular response under various physiological and pathological conditions [59]. It is generally accepted that ECs regulate vascular function in several ways; by immediate contact and by secreting signaling molecules or by transmitting electrical signals to VSMCs through myoendothelial gap junctions [60]. The membrane potential of VSMCs principally depends on the function of myoendothelial gap junctions [61]. Under *in vitro* circumstances, VSMCs are separated from the functional unit, and there is a lack of interactions between VSM and endothelium. Actually, the inconsistent expression of *α*- and *β*1-subunits of BK channels under pathological conditions has been reported by several studies previously. Downregulation of BK channels *β*1-subunits expression rather than *α*-subunits has been found in insulin-resistant rats [32]. Experimental diabetes induced reduced expression of BK *β*1-subunits while had no significant effects on *α*-subunits expression [21]. Besides, exposure of homocysteine reduced the BK *β*1-subunits expression without affecting the *α*-subunits expression in porcine coronary artery smooth muscle cells [62]. However, a clear understanding of this discordance and the underlying mechanisms is still lacking.

However, several gaps and limitations of this study need to be addressed in the future. First, the optimal dosage of Hyp requires clinical trials with large sample sizes. Second, the whole-cell K^+^ currents and single-channel currents should be further explored and validated. Third, further studies are necessary to probe the in-depth mechanisms. Furthermore, the inclusion of female rats may offer a more precise indication of the impact of Hyp pretreatment on I/R injury; however, our study only utilized male rats.

## 5. Conclusion

**Figure 9:**
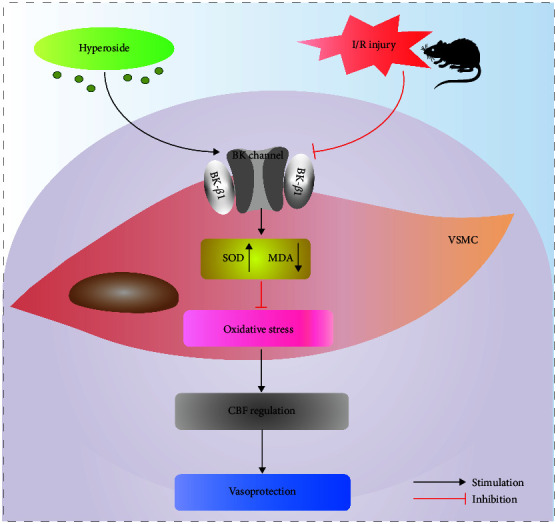
Diagram illustrates the potential actions of Hyp on BK channels against cerebral I/R injury. Hyp appears to upregulate BK *β*1-subunit expression in I/R-injured VSMCs. As a result of the effects of Hyp, the oxidative stress is inhibited, resulting in increases of CBF and vasoprotection in I/R-injured rats.

In summary, our results proved that Hyp had the therapeutic potential to prevent vascular outcomes, and the mechanism was associated with suppressing oxidative stress and improving CBF flux through activating BK channels. Besides this, we demonstrated that Hyp protected VSMCs against oxidative stress through upregulation of BK *β*1-subunits expression, indicating the importance of BK channels *β*1-subunits in vascular dysfunction (Figure 9). These findings may draw more attention to research into the therapeutic potential of Hyp in vascular pathophysiology in incidences of an ischemic stroke.

## Data Availability

If the magazine needs source data, it can be provided at any time.

## Abbreviations

Hyp: Hyperoside
BK: Large-conductance Ca^2+^-activated K+
I/R: Ischemia/reperfusion
OGD/R: Oxygen-glucose deprivation/reoxygenation
VSMCs: Vascular smooth muscle cells
MDA: Malondialdehyde
SOD: Superoxide dismutase
CBF: Cerebral blood flow
MCA: Middle cerebral artery
BA: Basal arteries
MCAO: Middle cerebral artery occlusion
IbTX: Iberiotoxin
ECs: Endothelial cells
ROI: Regions of interest.

## References

[1] Ko S.-B., Yoon B.-W. (2017). Blood pressure management for acute ischemic and hemorrhagic stroke: the evidence. *Seminars in Respiratory and Critical Care Medicine*.

[2] Marko M., Posekany A., Szabo S. (2020). Trends of r-tPA (recombinant tissue-type plasminogen activator) treatment and treatment-influencing factors in acute ischemic stroke. *Stroke*.

[3] Soares R. O. S., Losada D. M., Jordani M. C., Évora P., Castro-e-Silva O. (2019). Ischemia/reperfusion injury revisited: an overview of the latest pharmacological strategies. *International Journal of Molecular Sciences*.

[4] Chang L., Garcia-Barrio M. T., Eugene Chen Y. (2020). Perivascular adipose tissue regulates vascular function by targeting vascular smooth muscle cells. *Arteriosclerosis, Thrombosis, and Vascular Biology*.

[5] Zhou Z., Lu J., Liu W.-W. (2018). Advances in stroke pharmacology. *Pharmacology & Therapeutics*.

[6] Yang Y., Li J., Rao T., Fang Z., Zhang J. (2021). The role and mechanism of hyperoside against myocardial infarction in mice by regulating autophagy via NLRP1 inflammation pathway. *Journal of Ethnopharmacology*.

[7] Qiu J., Zhang T., Zhu X. (2020). Hyperoside induces breast cancer cells apoptosis via ROS-mediated NF-*k*B signaling pathway. *International Journal of Molecular Sciences*.

[8] Galeotti N. (2017). Hypericum perforatum (St John’s wort) beyond depression: a therapeutic perspective for pain conditions. *Journal of Ethnopharmacology*.

[9] Fan H.-H., Zhu L.-B., Li T. (2017). Hyperoside inhibits lipopolysaccharide-induced inflammatory responses in microglial cells via p38 and NF*κ*B pathways. *International Immunopharmacology*.

[10] Han N.-R., Go J.-H., Kim H.-M., Jeong H.-J. (2014). Hyperoside regulates the level of thymic stromal lymphopoietin through intracellular calcium signalling. *Phytotherapy Research*.

[11] Park J. Y., Han X., Piao M. J. (2016). Hyperoside induces endogenous antioxidant system to alleviate oxidative stress. *Journal of Cancer Prevention*.

[12] Guo X., Zhang Y., Lu C., Qu F., Jiang X. (2020). Protective effect of hyperoside on heart failure rats via attenuating myocardial apoptosis and inducing autophagy. *Bioscience, Biotechnology, and Biochemistry*.

[13] Fan Y.-F., Chen Z.-W., Guo Y., Wang Q.-H., Song B. (2011). Cellular mechanisms underlying Hyperin-induced relaxation of rat basilar artery. *Fitoterapia*.

[14] Ward R., Li W., Abdul Y. (2019). NLRP3 inflammasome inhibition with MCC950 improves diabetes-mediated cognitive impairment and vasoneuronal remodeling after ischemia. *Pharmacological Research*.

[15] Antigny F. (2019). Potassium channels in vascular smooth muscle: a pathophysiological and pharmacological perspective. *Fundamental & Clinical Pharmacology*.

[16] Kamata S., Kimura M., Ohyama S., Yamashita S., Shibukawa Y. (2021). Large-conductance calcium-activated potassium channels and voltage-dependent sodium channels in human cementoblasts. *Frontiers in Physiology*.

[17] Patel N. H., Johannesen J., Shah K. (2018). Inhibition of BK_Ca_ negatively alters cardiovascular function. *Physiological Reports*.

[18] Liu N., Yan F., Ma Q., Zhao J. (2020). Modulation of TRPV4 and BKCa for treatment of brain diseases. *Bioorganic & Medicinal Chemistry*.

[19] Xu H., Garver H., Fernandes R. (2015). BK channel *β*1-subunit deficiency exacerbates vascular fibrosis and remodelling but does not promote hypertension in high-fat fed obesity in mice. *Journal of Hypertension*.

[20] Borbouse L., Dick G. M., Asano S. (2009). Impaired function of coronary BK_Ca_ channels in metabolic syndrome. *American Journal of Physiology-Heart and Circulatory Physiology*.

[21] Li Y., Wang X.-L., Sun X. (2017). Regulation of vascular large-conductance calcium-activated potassium channels by Nrf2 signalling. *Diabetes & Vascular Disease Research*.

[22] Layne J. J., Nausch B., Olesen S.-P., Nelson M. T. (2010). BK channel activation by NS11021 decreases excitability and contractility of urinary bladder smooth muscle. *American Journal of Physiology-Regulatory, Integrative and Comparative Physiology*.

[23] Lin Y.-L., Dai Z.-K., Lin R.-J. (2010). Baicalin, a flavonoid from *Scutellaria baicalensis* Georgi, activates large-conductance Ca^2+^-activated K^+^ channels via cyclic nucleotide-dependent protein kinases in mesenteric artery. *Phytomedicine*.

[24] Yang Y., Lu F., Zhuang L. (2017). Combined preconditioning with hypoxia and GYKI-52466 protects rats from cerebral ischemic injury by HIF-1*α*/eNOS pathway. *American Journal of Translational Research*.

[25] Guo J.-M., Lin P., Duan J.-A., Shang E.-X., Qian D.-W., Tang Y.-P. (2012). Application of microdialysis for elucidating the existing form of hyperoside in rat brain: comparison between intragastric and intraperitoneal administration. *Journal of Ethnopharmacology*.

[26] Fan H., Li Y., Sun M. (2021). Hyperoside reduces rotenone-induced neuronal injury by suppressing autophagy. *Neurochemical Research*.

[27] Zuo Z., Zhang L., Zhou L., Chang Q., Chow M. (2006). Intestinal absorption of hawthorn flavonoids—in vitro, in situ and in vivo correlations.. *Life Sciences*.

[28] Li Q., Tian Z., Wang M. (2019). Luteoloside attenuates neuroinflammation in focal cerebral ischemia in rats via regulation of the PPAR*γ*/Nrf2/NF-*κ*B signaling pathway. *International Immunopharmacology*.

[29] Wen J.-Y., Zhang J., Chen S. (2021). Endothelium-derived hydrogen sulfide acts as a hyperpolarizing factor and exerts neuroprotective effects via activation of large-conductance Ca^2+^ -activated K^+^ channels. *British Journal of Pharmacology*.

[30] Chen J., Yu H., Zhong J. (2018). The phosphodiesterase-4 inhibitor, FCPR16, attenuates ischemia-reperfusion injury in rats subjected to middle cerebral artery occlusion and reperfusion. *Brain Research Bulletin*.

[31] Tang C., Xue H., Bai C., Fu R., Wu A. (2010). The effects of Tanshinone IIA on blood–brain barrier and brain edema after transient middle cerebral artery occlusion in rats. *Phytomedicine*.

[32] Salisbury E., Rodenberg E., Sonnet C. (2011). Sensory nerve induced inflammation contributes to heterotopic ossification. *Journal of Cellular Biochemistry*.

[33] Li X.-G., Wang Y.-B. (2019). SRPK1 gene silencing promotes vascular smooth muscle cell proliferation and vascular remodeling via inhibition of the PI3K/Akt signaling pathway in a rat model of intracranial aneurysms. *CNS Neuroscience & Therapeutics*.

[34] Tykocki N. R., Boerman E. M., Jackson W. F. (2017). Smooth muscle ion channels and regulation of vascular tone in resistance arteries and arterioles. *Comprehensive Physiology*.

[35] Ye Y., Gao M., Feng L., Feng B., Ma X. (2019). Isoliquiritigenin-induced vasodilation by activating large-conductance Ca^2+^ -activated K^+^ channels in mouse mesenteric arteries. *Clinical and Experimental Pharmacology and Physiology*.

[36] Steliga A., Kowianski P., Czuba E., Waśkow M., Moryś J., Lietzau G. (2020). Neurovascular unit as a source of ischemic stroke biomarkers—limitations of experimental studies and perspectives for clinical application. *Translational Stroke Research*.

[37] Tao T., Liu M., Chen M. (2020). Natural medicine in neuroprotection for ischemic stroke: challenges and prospective. *Pharmacology & Therapeutics*.

[38] Zhang Y.-M., Zhang Z.-Y., Wang R.-X. (2020). Protective mechanisms of quercetin against myocardial ischemia reperfusion injury. *Frontiers in Physiology*.

[39] Chen H., Wang J.-H., Ren Z.-X., Yang X.-B. (2006). Protective effect of hyperin on focal cerebral ischemia reperfusion injury in rats. *Zhong xi yi jie he xue bao = Journal of Chinese integrative medicine*.

[40] Ye P., Yang X.-L., Chen X., Shi C. (2017). Hyperoside attenuates OVA-induced allergic airway inflammation by activating Nrf2. *International Immunopharmacology*.

[41] Zemen B. G., Lai M. H., Whitt J. P., Khan Z., Zhao G., Meredith A. L. (2015). Generation of *Kcnma1^fl^-tdTomato*, a conditional deletion of the BK channel *α* subunit in mouse. *Physiological Reports*.

[42] Rueda A., Fernandez-Velasco M., Benitah J.-P., Gómez A. M. (2013). Abnormal Ca^2+^ Spark/STOC coupling in cerebral artery smooth muscle cells of obese type 2 diabetic mice. *PLOS ONE*.

[43] Hu X.-Q., Huang X., Xiao D., Zhang L. (2016). Direct effect of chronic hypoxia in suppressing large conductance Ca^2+^ -activated K^+^ channel activity in ovine uterine arteries via increasing oxidative stress. *The Journal of Physiology*.

[44] Andersson K.-E., Christ G. J., Davies K. P., Rovner E. S., Melman A. (2021). Gene therapy for overactive bladder: a review of BK-channel *α*-subunit gene transfer. *Therapeutics and Clinical Risk Management*.

[45] Kampa R. P., Sęk A., Szewczyk A., Bednarczyk P. (2021). Cytoprotective effects of the flavonoid quercetin by activating mitochondrial BKCa channels in endothelial cells. *Biomedicine & Pharmacotherapy*.

[46] Wang N., He D., Zhou Y. (2020). Hydroxysafflor yellow a actives BK_Ca_ channels and inhibits L-type Ca channels to induce vascular relaxation. *European Journal of Pharmacology*.

[47] Chao J., Guo Y., Chao L. (2018). Protective role of endogenous kallistatin in vascular injury and senescence by inhibiting oxidative stress and inflammation. *Oxidative Medicine and, Cellular Longevity*.

[48] Saha P. S., Knecht T. M., Arrick D. M., Watt M. J., Scholl J. L., Mayhan W. G. (2023). Prenatal exposure to alcohol impairs responses of cerebral arterioles to activation of potassium channels: role of oxidative stress. *Alcoholism, Clinical and Experimental Research*.

[49] Coskun C., Tokgun O. (2021). BK channel opener protects cell viability by regulating reactive oxygen levels in astrocyte cells. *General Physiology and Biophysics*.

[50] Hritcu L., Ionita R., Postu P. A. (2017). Antidepressant flavonoids and their relationship with oxidative stress. *Oxidative Medicine and Cellular Longevity*.

[51] Guo Y., Yu X.-M., Chen S., Wen J.-Y., Chen Z.-W. (2020). Total flavones of *Rhododendron simsii* planch flower protect rat hippocampal neuron from hypoxia-reoxygenation injury via activation of BK_Ca_ channel. *Journal of Pharmacy and Pharmacology*.

[52] He J., Li H., Li G., Yang L. (2019). Hyperoside protects against cerebral ischemia-reperfusion injury by alleviating oxidative stress, inflammation and apoptosis in rats. *Biotechnology & Biotechnological Equipment*.

[53] Bollinger W. L., Sial N., Dawson-Scully K. (2018). BK channels and a cGMP-dependent protein kinase (PKG) function through independent mechanisms to regulate the tolerance of synaptic transmission to acute oxidative stress at the *Drosophila* larval neuromuscular junction. *Journal of Neurogenetics*.

[54] Slupe A. M., Kirsch J. R. (2018). Effects of anesthesia on cerebral blood flow, metabolism, and neuroprotection. *Journal of Cerebral Blood Flow and Metabolism*.

[55] Chanda D., Prieto-Lloret J., Singh A. (2016). Glabridin-induced vasorelaxation: evidence for a role of BK_Ca_ channels and cyclic GMP. *Life Sciences*.

[56] Hong Z., Reeves K. J., Sun Z., Li Z., Brown N. J., Meininger G. A. (2015). Vascular smooth muscle cell stiffness and adhesion to collagen i modified by vasoactive agonists. *PLOS ONE*.

[57] Li Y., Wang X.-L., Sun X. (2017). Regulation of vascular large-conductance calcium-activated potassium channels by Nrf2 signalling. *Diabetes and Vascular Disease Research*.

[58] Navarro-Antolin J., Levitsky K. L., Calderon E., Ordóñez A., López-Barneo J. (2005). Decreased expression of maxi-K^+^ channel *β*_1_ -subunit and altered vasoregulation in hypoxia. *Circulation*.

[59] Mendez-Barbero N., Gutierrez-Munoz C., Blanco-Colio L. M. (2021). Cellular crosstalk between endothelial and smooth muscle cells in vascular wall remodeling. *International Journal of Molecular Sciences*.

[60] Makino A., Firth A. L., Yuan J. X.-J. (2011). Endothelial and smooth muscle cell ion channels in pulmonary vasoconstriction and vascular remodeling. *Comprehensive Physiology*.

[61] Hald B. O., Jacobsen J. C. B., Sandow S. L., Holstein-Rathlou N.-H., Welsh D. G. (2014). Less is more: minimal expression of myoendothelial gap junctions optimizes cell-cell communication in virtual arterioles. *Journal of Vascular Research*.

[62] Sun W.-T., Wang X.-C., Novakovic A., Wang J., He G.-W., Yang Q. (2019). Protection of dilator function of coronary arteries from homocysteine by tetramethylpyrazine: role of ER stress in modulation of BK_Ca_ channels. *Vascular Pharmacology*.

